# An explainable self-attention deep neural network for detecting mild cognitive impairment using multi-input digital drawing tasks

**DOI:** 10.1186/s13195-022-01043-2

**Published:** 2022-08-09

**Authors:** Natthanan Ruengchaijatuporn, Itthi Chatnuntawech, Surat Teerapittayanon, Sira Sriswasdi, Sirawaj Itthipuripat, Solaphat Hemrungrojn, Prodpran Bunyabukkana, Aisawan Petchlorlian, Sedthapong Chunamchai, Thiparat Chotibut, Chaipat Chunharas

**Affiliations:** 1 Computational Molecular Biology Group, Faculty of Medicine, Chulalongkorn University, Bangkok, 10330 Thailand; 2 National Nanotechnology Center, National Science and Technology Development Agency, Pathum Thani, 12120 Thailand; 3 Center for Artificial Intelligence in Medicine, Research Affairs, Faculty of Medicine, Chulalongkorn University, Bangkok, 10330 Thailand; 4 Neuroscience Center for Research and Innovation, Learning Institute, King Mongkut’s University of Technology Thonburi, Bangkok, 10140 Thailand; 5 Big Data Experience Center, King Mongkut’s University of Technology Thonburi, Bangkok, 10140 Thailand; 6 Chula Neuroscience Center, King Chulalongkorn Memorial Hospital, Thai Red Cross Society, Bangkok, 10330 Thailand; 7 Department of Psychiatry, Faculty of Engineering, Chulalongkorn University, Bangkok, 10330 Thailand; 8 Department of Computer Engineering, Faculty of Engineering, Chulalongkorn University, Bangkok, 10330 Thailand; 9 Geriatric Excellence Center, King Chulalongkorn Memorial Hospital, Thai Red Cross Society, Faculty of Medicine, Chulalongkorn University, Bangkok, 10330 Thailand; 10 Cognitive Clinical and Computational Neuroscience, Department of Internal Medicine, Faculty of Medicine, Chulalongkorn University, Bangkok, 10330 Thailand; 11 Chula Intelligent and Complex Systems Lab, Department of Physics, Faculty of Science, Chulalongkorn University, Bangkok, 10330 Thailand

## Abstract

**Background:**

Mild cognitive impairment (MCI) is an early stage of cognitive decline which could develop into dementia. An early detection of MCI is a crucial step for timely prevention and intervention. Recent studies have developed deep learning models to detect MCI and dementia using a bedside task like the classic clock drawing test (CDT). However, it remains a challenge to predict the early stage of the disease using the CDT data alone. Moreover, the state-of-the-art deep learning techniques still face black box challenges, making it questionable to implement them in a clinical setting.

**Methods:**

We recruited 918 subjects from King Chulalongkorn Memorial Hospital (651 healthy subjects and 267 MCI patients). We propose a novel deep learning framework that incorporates data from the CDT, cube-copying, and trail-making tests. Soft label and self-attention were applied to improve the model performance and provide a visual explanation. The interpretability of the visualization of our model and the Grad-CAM approach were rated by experienced medical personnel and quantitatively evaluated using intersection over union (IoU) between the models’ heat maps and the regions of interest.

**Results:**

Rather than using a single CDT image in the baseline VGG16 model, using multiple drawing tasks as inputs into our proposed model with soft label significantly improves the classification performance between the healthy aging controls and the MCI patients. In particular, the classification accuracy increases from 0.75 (baseline model) to 0.81. The F1-score increases from 0.36 to 0.65, and the area under the receiver operating characteristic curve (AUC) increases from 0.74 to 0.84. Compared to the multi-input model that also offers interpretable visualization, i.e., Grad-CAM, our model receives higher interpretability scores given by experienced medical experts and higher IoUs.

**Conclusions:**

Our model achieves better classification performance at detecting MCI compared to the baseline model. In addition, the model provides visual explanations that are superior to those of the baseline model as quantitatively evaluated by experienced medical personnel. Thus, our work offers an interpretable machine learning model with high classification performance, both of which are crucial aspects of artificial intelligence in medical diagnosis.

## Introduction

Approximately 50 million people are currently suffering from dementia worldwide [1]. Unfortunately, there is no cure for such a devastating condition. One of the most crucial management strategies for preventing the disease from progressing is to detect the initial stage of pathological cognitive aging as early as possible. This initial stage of cognitive decline is known as mild cognitive impairment (MCI), which has a prevalence rate of about 20% of the elderly population aged 60 and above [2–4].

The clock drawing test (CDT) is one of the most studied neuropsychological tests known for its ability to capture a wide range of neurocognitive disorders including Alzheimer’s disease and other types of dementia. Accordingly, it has been included in a rapid population-based screening test for dementia [5]. While the CDT could be easily implemented in the pen-and-paper format, it still requires highly trained medical personnel to administer the screening as well as analyze and interpret the test results. Recently, research studies have tried to overcome these limitations by collecting the data in digital format and adopting advanced machine learning (ML) models to automate and improve the scoring and disease classification methods [6–16]. Initially, early ML research has demonstrated promising classification results obtained from domain knowledge feature construction guided by human experts [6–8, 11, 15, 16]. Recent studies use deep learning models to avoid the need for such hand-crafted features and improve the performances in several neuropsychological tests including digit classification [9], digit-and-clock-hand recognition [10], contour-and-hand segmentation and digit classification [12], clock score prediction [13], and healthy-versus-cognitive-impaired classification [14].

In contrast to the case of dementia, the success in using digital clock drawing and deep learning to detect less severe neurocognitive disorders like MCI is still limited. To improve the model performance, it is possible to combine multiple drawing tasks such as a trail-making test and a copy-drawing test as inputs to a deep learning model. Indeed, a prior study recently demonstrated different classification accuracies when different drawing tasks were used [17]. However, deep learning is often referred to as a black box approach given that most of deep learning models provide only predictions without explanation that can be understood by humans. Therefore, these deep learning models are usually not applicable in clinical settings where the predictions are expected to be interpretable by healthcare providers [18].

In this work, we developed a novel multi-input deep learning model that integrates three different drawing tasks to perform an explainable MCI detection. Extending clock drawing-based detection with deep learning [9, 10, 12–14] to include a cube-copying drawing and a trail-making test into model inputs, our convolutional neural network (CNN) equipped with the self-attention mechanism (multi-input Conv-Att) achieves an excellent classification performance. The multi-input Conv-Att model enjoys an improvement of 0.051, 0.241, and 0.095 gain on the average accuracy, F1-score, and area under the receiver operating characteristic curve (AUC), respectively, compared to those of a baseline CNN. While the prediction accuracy of the multi-input Conv-Att model is comparable to that of the baseline CNN with Grad-CAM [19], the multi-input Conv-Att model provides visual cues that are more consistent with how clinical experts analyze drawing tasks. We also examine the standard medical criterion for MCI diagnosis—the drawings are classified as MCI with certainty if their score drops below a hard cutoff. Our data reveal that the scores of the healthy population and those of the MCI population strongly overlap, especially near the cutoff. Thus, we demand our multi-input Conv-Att model to output a class probability (i.e., soft labels), rather than a class with certainty, to accommodate the classification uncertainty near the cutoff. With soft labels, we further gain an improvement of 0.013 and 0.056 on the average accuracy and F1-score. We have made our dataset publicly available for interested researchers to benchmark their methods at https://github.com/cccnlab/MCI-multiple-drawings.

## Results

We assessed the MCI vs. healthy aging control classification performance of our proposed method, multi-input Conv-Att with a soft label, on a dataset of 918 subjects (138 of which were used as unseen test data: 98 healthy aging controls and 40 MCI patients) acquired with informed consents at King Chulalongkorn Memorial Hospital, Bangkok, Thailand (see the “Materials and methods” section for more details). Each subject was categorized based on a Montreal Cognitive Assessment (MoCA) score cutoff of 25 [20], resulting in 651 healthy subjects and 267 MCI patients in the dataset. We compared the proposed method to a baseline model, denoted as single-input VGG16 with only the clock drawing test, that is closely related to existing deep learning-based methods [13, 14] on MCI vs. healthy aging control classification in terms of the framework used: input data, deep learning components, and a training procedure.

**Fig. 1:**
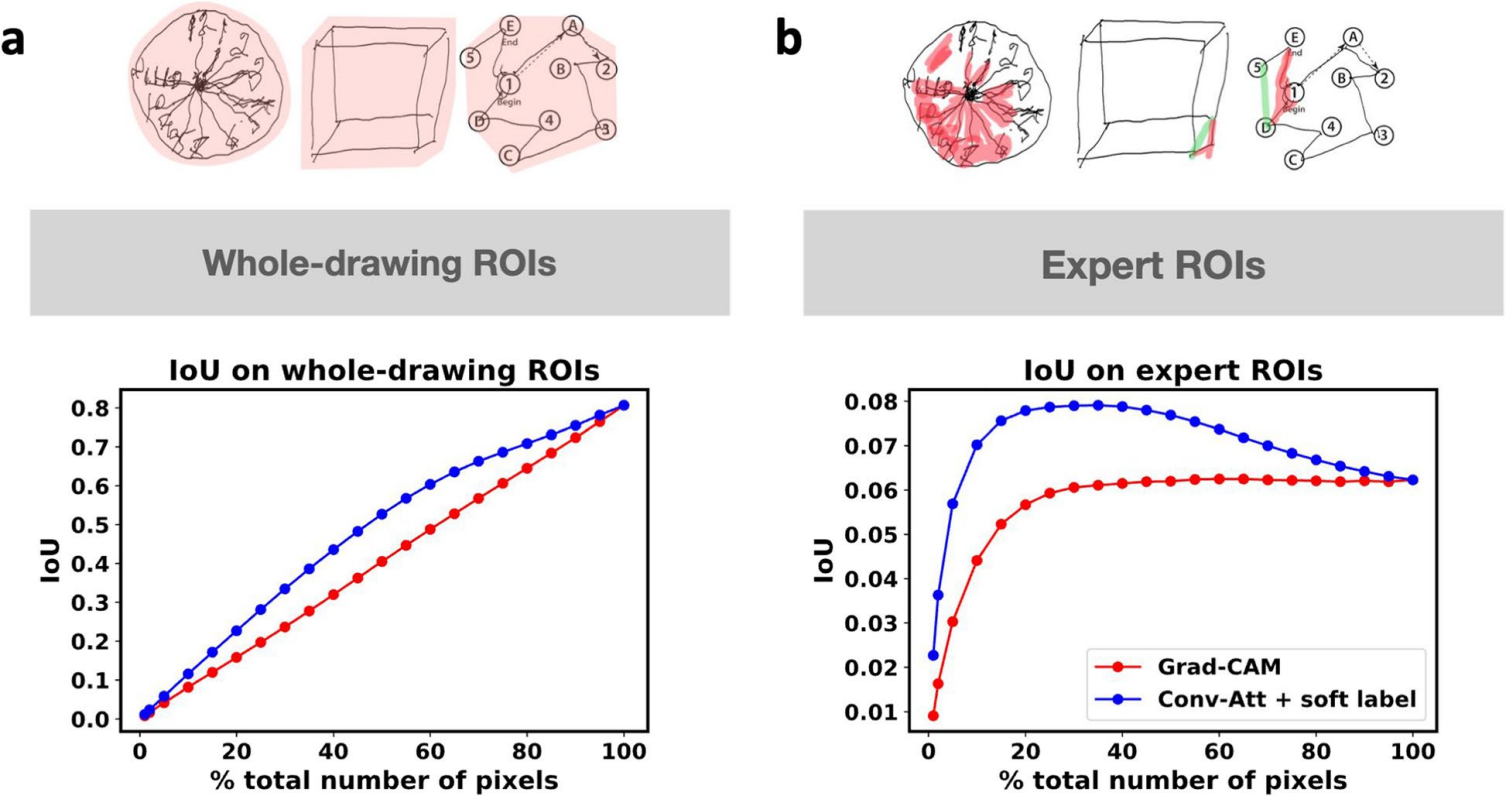
Quantitative comparisons between the multi-input VGG16 model with Grad-CAM (red) and our multi-input Conv-Att model with soft label (blue), as measured by the IoUs between the heat maps and two types of ROIs, (**a**) whole-drawing ROIs and (**b**) expert ROIs, as a function of the percentage of the number of pixels used in the heat maps. Example images with corresponding ROIs are shown at the top of each panel. Our proposed model is more similar to both whole-drawing and expert ROIs than Grad-CAM model and the higher similarity is consistent over broad range of % total number of pixels from the models’ outputs (10%-80%)

We reported the classification results in Table 1 and assessed the ability of the proposed method to support its classification decisions (MCI vs. healthy aging control) through visual interpretability. In particular, we demonstrated that the proposed method yielded improved heat maps compared to those generated by the multi-input VGG16 model with Grad-CAM visualization [19], as measured by two metrics: (1) the interpretability scores given by 3 experts (a neurologist and two licensed neuropsychologists) (Table 2) and (2) the intersection over union (IoU) between the heat maps obtained from each method and the corresponding ground truth regions-of-interest (ROIs) (Fig. 1).

**Table 1:** The mean and standard deviation of the classification accuracies, F1-scores, and AUCs over 5 different random training-validation-test data splittings. Our proposed model, which benefits from the incorporation of multiple complementary drawing tasks (clock drawing, cube-copying, and trail-making), self-attention mechanism, and soft labeling approach, achieved much higher mean accuracy, F1-score, and AUC than the baseline model

Models	Accuracy	F1-score	AUC
VGG16 with only clock-drawing test	0.7478 ± 0.0071	0.3573 ± 0.0443	0.7429 ± 0.0131
VGG16 with only cube-copying test	0.7739 ± 0.0096	0.4994 ± 0.0477	0.7813 ± 0.0197
VGG16 with only trail-making test	0.7739 ± 0.0249	0.5283 ± 0.0548	0.7722 ± 0.0240
Multi-input VGG16	0.7986 ± 0.0071	0.5938 ± 0.0207	0.8115 ± 0.0192
Conv-Att with only clock-drawing test	0.7522 ± 0.0125	0.3586 ± 0.0309	0.7337 ± 0.0204
Conv-Att with only cube-copying test	0.7768 ± 0.0168	0.5095 ± 0.0515	0.7791 ± 0.0199
Conv-Att with only trail-making test	0.7696 ± 0.0167	0.5211 ± 0.0272	0.7662 ± 0.0231
Multi-input Conv-Att	0.7986 ± 0.0071	0.5981 ± 0.0221	0.8379 ± 0.0176
**Multi-input Conv-Att with a soft label (proposed)**	**0.8116** ***±*** **0.0103**	**0.6539** ***±*** **0.0097**	**0.8375** ***±*** **0.0116**

**Table 2:** The mean and standard deviation of the visual interpretability scores over all samples in the test set given by a neurologist and two licensed neuropsychologists (scores from 1 to 5; 1 being the worst and 5 being the best in terms of providing a visual interpretability that aligned with their experience and knowledge)

Evaluators	VGG16 with Grad-CAM	Conv-Att with a soft label (proposed)
Expert 1	1.42 ± 0.74	3.41 ± 0.61
Expert 2	1.86 ± 0.74	2.20 ± 0.93
Expert 3	1.36 ± 0.62	2.87 ± 0.68

Since the proposed method strongly deviates from existing works by the incorporation of three specially designed components, which consist of (1) the multi-input approach (clock drawing, cube-copying, and trail-making inputs), (2) self-attention mechanism, and (3) soft labeling technique, we additionally performed an ablation study to determine the relative improvement gained from each of the proposed components, as quantitatively measured by the classification accuracies, F1-scores, and AUC.

### MCI vs. healthy aging control classification

As shown in Table 1, the proposed method yielded the mean classification accuracy of 0.8116, F1-score of 0.6539, and AUC of 0.8375 over five repetitions, demonstrating 8.53%, 83%, and 12.7% relative improvements in the mean accuracy, F1-score, and AUC, respectively, with respect to the baseline method. By extending the single-input models (VGG16 and Conv-Att) to their corresponding multi-input models (multi-input VGG16 and multi-input Conv-Att), we observed significant improvements in the classification accuracies, AUCs, and, more remarkably, F1-scores. While the classification accuracies, F1-scores, and AUCs obtained from VGG16 and Conv-Att were comparable in both the single-input and multi-input cases, the self-attention mechanism included in the Conv-Att models resulted in improved visual interpretability which will be discussed in the next subsection in detail. With the addition of the soft labeling technique, both accuracy and F1-score of the proposed multi-input Conv-Att model further improved.

### Visual interpretability

**Fig. 2:**
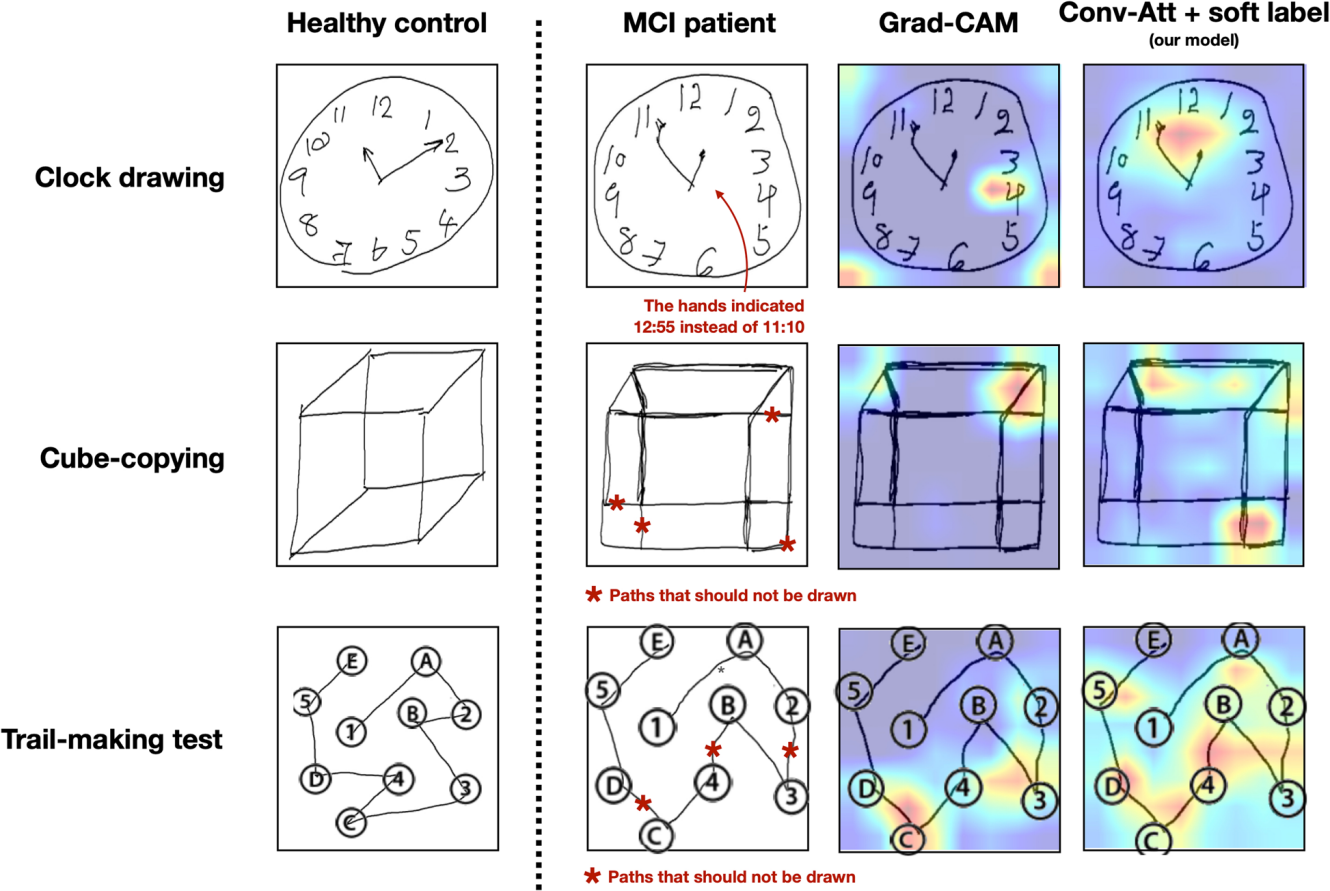
Visual explanations provided by the multi-input VGG16 model with Grad-CAM visualization (2^nd^ column from the right) and the proposed model (column on the far-right) on a representative MCI test sample (2^nd^ column from the left). For the clock image (1^st^ row), our model highlights the hands of the clock where it says 12:55 instead of 11:10. For the cube-copying image (2^nd^ row), our model highlights unusual paths better. For the trail-making test (last row), our model could focus along the paths that should not have been drawn (paths from 2-3, B-4 and C-D), while the multi-input VGG16 model with Grad-CAM failed to highlight some of those paths (B-4). Note that the red arrow and asterisks were not drawn by the subjects but added here to aid the descriptions

An example of visual interpretability of the multi-input VGG16 model with Grad-CAM [19] and the proposed model is shown in Fig. 2. The visual interpretability scores given by the three experts are summarized in Table 2. The proposed method was given higher average interpretability scores by all the experts, indicating that the heat maps generated by our method were better aligned with the experts’ clinical experience, compared to those of the multi-input VGG16 model with Grad-CAM. Furthermore, as shown in Fig. 1, the heat maps generated by our proposed method yielded significantly higher average IoUs between the heat maps and the ground truth ROIs than those of the multi-input VGG16 model with Grad-CAM.

## Discussion

By incorporating three specially designed components into a standard CNN, consisting of the multi-input approach, self-attention mechanism, and soft labeling technique, our proposed model outperformed the baseline CNN model as measured by not only several quantitative evaluation metrics (accuracy, F1-score, and AUC), but also the visual explanation provided by the model’s heat maps. Unlike the previous studies that take only a clock drawing image as input to the model [13, 14], our proposed multi-input model exploits the complementary information provided by the three drawing tasks that rely on different combinations of fundamental cognitive abilities under the neuropsychological perspective: planning and task-switching in the trail-making test, visuospatial ability in the cube-copying drawing test, and both planning and visuospatial ability in the clock drawing test. This approach can be considered as an extension of previous research that achieved different accuracies when different drawing tasks were used [17]. By replacing the global max-pooling layer of a standard CNN architecture with a stack of self-attention layers, the proposed model was able to represent the data more efficiently and provide improved heat maps that could be used to support the model’s classification decision, compared to the baseline model visualized using Grad-CAM [19]. In addition to the multi-input and self-attention components, the proposed model benefits from the soft labeling technique that takes into account the uncertainty of the diagnostic labels (i.e., MCI vs. healthy aging control) near the MoCA score cutoff, resulting in further improvement in the classification performance.

While typical model evaluation tends to focus on classification performance, there are some other important aspects of what counts as a good model. In our case, we found that even though applying self-attention did not lead to better classification performance, clinical experts preferred the visual explanation provided by our model to those of multi-input VGG16. The clinical experts found that our model was able to highlight the areas that aligned with their experience and knowledge. For example, our model was able to highlight the clock hands where the locations of the short and long hands were incorrectly drawn (see Fig. 2). Moreover, the heat maps of our proposed model were better aligned with the ground truth ROIs (Fig. 1), as indicated by higher IoUs.

Having a machine learning model that can provide interpretable outputs instead of only providing diagnostic probability is especially important in a medical condition with mixed etiologies like MCI. Two MCI patients might draw a clock, a cube, and trails differently because their MCIs have different underlying pathological mechanisms. This capability will be beneficial to healthcare personnel since they can synergistically combine such interpretable outputs with their clinical judgments to potentially gain more insight into the underlying mechanism of MCI. This improved interpretability will become even more critical when machine learning is used as a decision support system—the direction that is gaining more attention.

In this study, we benchmarked our proposed model to not only a strong VGG16 baseline, but also several extensions of the baseline through our ablation study. Nevertheless, it is not straightforward to compare our reported quantitative metrics to those reported in existing works due to many reasons. First, different proxies of neurocognitive disorders have been adopted in different studies. For example, while the Shulman clock scoring system [21] has been used as a surrogate marker for dementia [13], we used Petersen’s criteria with the MoCA score cutoff of 25 [4, 20] for the MCI diagnosis in our study. Moreover, different study populations with potentially different demographic information and recruiting procedures were involved. Such experimental inconsistencies make it difficult for direct comparisons between the studies (for example, our data came from our healthy geriatric clinic where the MCI patients have only mild symptoms), warranting the sharing of the datasets of each study for better benchmarking. Consequently, we have made our dataset publicly available at https://github.com/cccnlab/MCI-multiple-drawings.

It is straightforward to extend our model to add other drawing tests since an image from each test is processed by its own VGG16 model and a stack of self-attention layers (called a feature extraction pathway), and the extracted features from all the tests are combined at the last layer of our model. For example, to accommodate additional drawing tests, more feature extraction pathways can be included in our model: one pathway per test. The extracted features from all the pathways can then be combined using the concatenation operation. To retrain the model, the parameters of the already-trained pathways can be used to initialize those in the newly added pathway(s). Additional tests added may help alleviate the impaired domains of existing tests and improve the accuracy of the overall model.

Switching the MoCA test from pen-and-paper to the digital format allows us to store the drawing trajectory of each drawing task, which contains both temporal and spatial information. While the proposed model, which only uses the final drawing as its input (only the spatial information) and discards the information-rich temporal information provided by the drawing trajectory, already achieved much higher accuracy, F1-score, and AUC than those of the baseline model, we project that its extension to a spatio-temporal version would further improve the classification performance with the presence of a larger amount of data. Since there exist many drawing trajectories that correspond to the same final drawing, having access to the raw drawing trajectory would enable the model to come up with potentially better data representation in the spatio-temporal domain. Moreover, exploring hidden structures in the high-dimensional space of raw drawing trajectory using unsupervised learning is also an interesting future direction.

### Limitations of the study

Although our sample size is relatively large compared to previous studies because we did not specifically screen for MCI cases, the fraction of the MCI patients in the dataset is low (29%, 267 out of 918 subjects). Moreover, our subjects tend to receive higher levels of education than the national average and consist of a relatively high female proportion. These factors could influence our model and should be considered when applying our work to a different population.

The diagnosis of MCI in our study is based on Petersen’s criteria which is a standard clinical practice. However, some research studies added additional biomarkers such as cerebrospinal fluid (CSF) total tau protein, phosphorylated tau protein, Aβ42, and Positron emission tomography (PET) to the clinical criteria. We did not include the biomarkers in this study because it would reduce the number of subjects even further, and having sufficient data is critical for developing deep learning models.

## Conclusion

In summary, we found that, in a challenging scenario where the aim is to identify MCI patients among healthy aging controls, using multiple inputs to train the model with soft labels and the self-attention mechanism leads to substantial improvements in model performance. The visual explanation provided by our proposed model is superior to the baseline model as rated by experienced medical personnel and quantitatively evaluated using the IoU between the models’ heat maps and ROIs. Thus, our model yields better classification performance and interpretability—both of which are critical aspects of the future development of artificial intelligence in medical diagnosis.

## Materials and methods

### Data collection

Under the institutional review board approval, a digital version of the MoCA test was administered on a tablet with a digital pen to a total of 918 subjects with informed consents by trained psychologists at King Chulalongkorn Memorial Hospital, Bangkok, Thailand. The population came from a healthy elderly cohort which focused on preventive care for healthy Thai citizens without major medical conditions (such as organ failures). The median age was 67 years old (ranging from 55 to 89 years old), 77% female, 44% received bachelor’s degree, and 20% received higher education. For the clock drawing task, the subjects were instructed to “draw a circular clock face with all the numbers and clock hands indicating the time of 10 min past 11 o’clock.” In the cube-copying test, the subjects were instructed to copy the Necker cube image on an empty space. In the trail-making test, the subjects were instructed to “draw a line that goes from a number to a letter in an ascending order, starting at number 1 (pointing to the number 1), to this letter (pointing to the letter A), then go to the next number (pointing to the number 2), and so on.”

For each subject, we extracted the drawn clock drawing, cube-copying, and trail-making images along with the MoCA score. We then categorized the subjects into healthy aging controls and MCI patients based on their MoCA scores. In particular, the subjects were categorized as having MCI if the MoCA scores were below 25 as typically used in clinical routines [20], resulting in 651 healthy subjects and 267 MCI patients in our dataset. The collected data were randomly split into three groups in a stratified fashion: 70% as training, 15% as validation, and 15% as test data. All images were resized to 256 × 256 in all experiments.


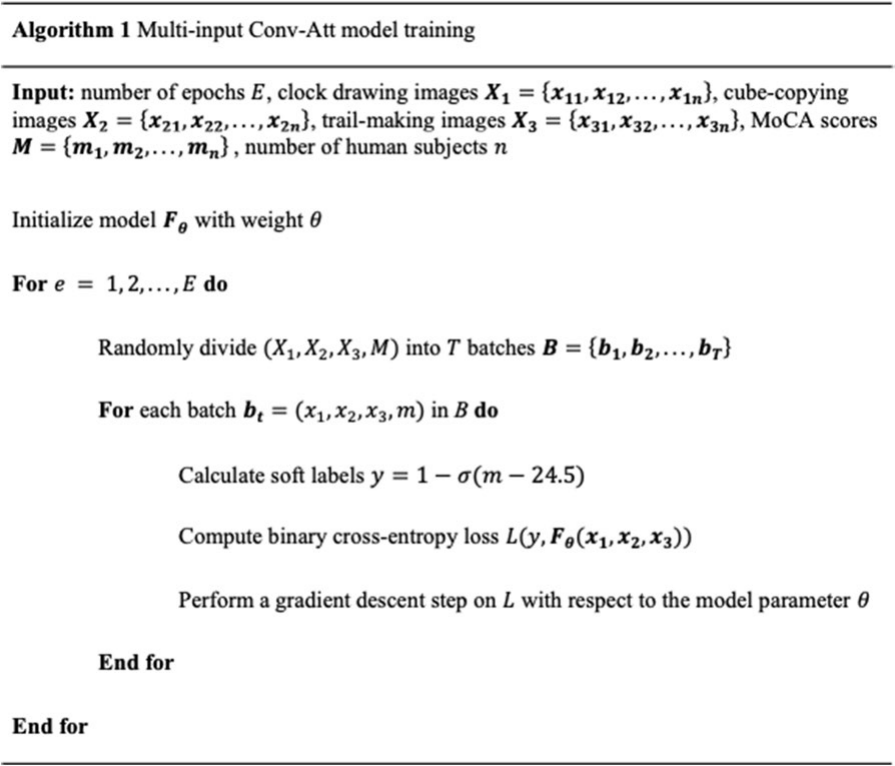


### Proposed method: multi-input Conv-Att model with soft labels

**Fig. 3:**
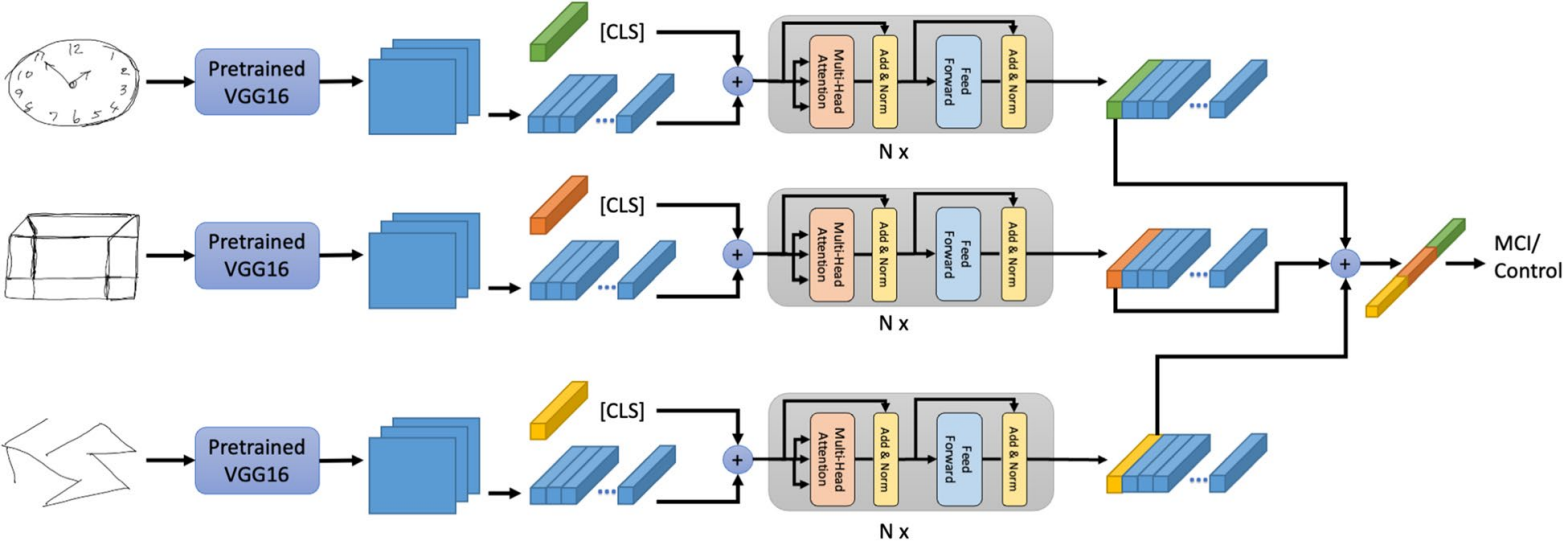
Overview of our proposed multi-input Conv-Att model. Our model simultaneously takes clock drawing, cube-copying, and trail-making images as its inputs and processes them using a cascade of CNNs and a stack of self-attention layers

We developed a multi-input deep learning method for MCI vs healthy aging control classification that is a cascade of CNN backbones and self-attention layers [22, 23] trained with soft labels, as shown in Fig. 3. As opposed to existing models which take a clock drawing image as the only input to the models [13, 14], our proposed multi-input model takes clock drawing, cube-copying, and trail-making images simultaneously as inputs, exploiting complementary information offered by the three neuropsychological tests. Incorporating the self-attention layers into the model leads to more efficient image representations, compared to typical CNNs, that can later be used to support the model’s classification decision through heat map visualization. The soft label component of our method is designed to aid our model training by taking into account the uncertainty of the diagnostic labels (i.e., MCI vs. healthy aging control) near the designated MoCA score cutoff. An overview of the training process of the proposed method is presented in Algorithm 1. In the following subsections, we described each of the components in detail.

#### Conv-Att model architecture

As shown in Fig. 3, clock drawing, cube-copying, and trail-making images are used as inputs to our model. Each of the three images is passed into a separate CNN backbone (VGG16 [24] pretrained on the ImageNet dataset [25], followed by a stack of self-attention layers, resulting in a vectorized image representation). Including the self-attention layers in the model leads to not only efficient image representation, but also improved visual explanation for MCI vs. healthy aging control classification. Then, the resulting vectors from the three tasks are concatenated and processed by a two-node fully connected layer with the softmax function ${f}_i\left(\overrightarrow{x}\right)=\frac{e^{x_i}}{\sum_j{e}^{x_j}}$.

#### Self-attention

Unlike in a standard image classification model, we employ self-attention instead of a pooling layer to aggregate the output from the CNN backbone,﻿ $\widetilde X\in\mathbb{R}^{H\times L\times C}$.,and *C* are the height, width, and number of filters, respectively. First, we initialize a random classification token vector, ﻿[CLS] $\in\mathbb{R}^D$ where *D* is the hidden dimension in the self-attention mechanism used in BERT [26]. The [CLS] vector is used to aggregate visual representation from all pixels in *X*. Second, 1 × 1 convolution with *D* output filters are applied to *X* to adjust its last dimension to match the hidden dimension *D* of the [CLS] vector. After that, it is reshaped into a matrix of shape *HL* × *D* and concatenated with [CLS] resulting in ﻿$\overset{\sim }{X}\in {\mathbb{R}}^{\left( HL+1\right)\times D}$. Reshaping the data this way enables us to investigate how much each pixel contributes to the final classification decision through the attention rollout method [27]. The self-attention is defined as:

$$\mathrm{Attn}\left(Q,K,V\right)=\mathrm{softmax}\left(\frac{Q{K}^T}{\sqrt{D}}\right)V$$

where﻿ *Q*, *K*, *V* $\in\mathbb{R}$^D × *D*^ are the query, key, and value, respectively, which are the projections of $\overset{\sim }{X}\in {\mathbb{R}}^{\left( HL+1\right) xD}$ with different linear functions: $Q=\overset{\sim }{X}{W}_Q^T,K=\overset{\sim }{X}{W}_K^T,$ and $V=\overset{\sim }{X}{W}_V^T$ where﻿ *W*_*Q*_, *W*_*K*_, *W*_*V *_ $\in\mathbb{R}$^*D × D*^. At the final layer of self-attention, the vector at the [CLS] position is used as the final image representation.

#### Soft-label

As explained in the “Data collection” section, we assigned the label of 0 (healthy control) to a subject with the MoCA score higher than or equal to 25, and the label of 1 (MCI) otherwise. Such a labeling approach is typically referred to as hard labeling. While training a model with hard labels is the most commonly used approach to solving binary classification, we propose to train our proposed model with soft labels based on MoCA scores for MCI vs. healthy aging control classification to take into account the uncertainty of the diagnostic labels (MCI vs. healthy aging control) near the MoCA score cutoff of 25. Specifically, we define a soft label *y* according to the following equation:

$$y=1-\sigma \left(m-24.5\right)$$

where *m* is a MoCA score, and *σ* denotes the sigmoid function. Since a subject with the MoCA score of 24 is labeled as an MCI patient and a subject with the MoCA score of 25 is labeled as a healthy control, we subtract 24.5 from *m* so that the center of the sigmoid will be at 24.5.

The hard threshold of 25, below which a subject is considered an MCI patient, is a man-made criterion, rather than the number revealed through rigorous statistical tests from a large number of trials and can be varied depending on contexts such as education or cultures [20, 28, 29]. Rather, by assigning a soft label, the uncertainty in the classification result is manifested through the sigmoidal probability function. So, in a post hoc way, the soft label approach can help relax the strong classification bias inherent in the hard label approach. We trained the proposed model by minimizing the binary cross-entropy loss:

$$L=-\frac{1}{M}{\sum}_{i=1}^M\left({y}_i\log {p}_i+\left(1-{y}_i\right)\log \left(1-{p}_i\right)\right)$$

where *M* is the number of training data, *y*_*i*_ is the soft label of the data *i*, and *p*_*i*_ is the output of the model which can be interpreted as the predicted probability that the data *i* is associated with MCI.

#### Attention rollout

To visualize how self-attention combines the pixels of the last feature maps calculated by the CNN backbone *X* into the final image representation, we used attention rollout [27]. In the self-attention layers, there exist residual connections between consecutive layers. Therefore, the output of the self-attention layer *l* + 1 is defined below:

$${V}_{l+1}={V}_l+{W}_{att}{V}_l$$

where *W*_*att*_ is the attention weight, and *V*_*l*_ is the output of the self-attention layer *l*. To compensate for the effects of the residual connections, the raw attention *A* is *A* = 0.5*W*_*att*_ + 0.5*I* where *I* is the identity matrix. The attentions from the self-attention layer *l*_*i*_ to layer *l*_*j*_ are computed by recursively multiplying the attention weights as follows:

$$\widetilde A\left(l_i\right)=\left\{\begin{array}{lc}A\left(l_i\right)\widetilde A\left(l_{i-1}\right),&i>j\\\;\;\;\;\;\;\;\;\;\;\;\;A\left(l_i\right),&i=j\end{array}\right.$$

where $\overset{\sim }{A}\left({l}_i\right)$ is the attention rollout at the self-attention layer *l*_*i*_, and *A*(*l*_*i*_) is the raw attention at the self-attention layer *l*_*i*_. The interpretability from our model is how the self-attention layers combine the last feature maps into the final image representation. Therefore, it is equivalent to the attention rollout for [CLS] over all the pixels of the last feature map. The attention rollout for each [CLS] is reshaped back to the size of the last feature map and then resized to match the size of the original input image. The heat map from each [CLS] is used as the interpretability for each input image.

### Experiments

We compared our proposed method to four VGG16-based models: single-input VGG16 models that take only an image from one of the three tasks (i.e., clock drawing, cube-copying, and trail-making) as input and a multi-input VGG16 model that simultaneously takes clock drawing, cube-copying, and trail-making images as inputs. For the multi-input version, different VGG16s were used to process different input images. At the end of each VGG16, the global average pooling layer was applied. The average pooled image features from each task were concatenated and then passed into a two-node fully connected layer with the softmax function.

We also compared the results of the proposed method to those of the proposed method with some components removed. In particular, we recorded the performances of the single-input Conv-Att models and the multi-input Conv-Att model, both trained with hard labels.

#### Model training

Adam [30] optimizer with the learning rate of 1e−5, *β*_1_ = 0.9, *β*_2_ = 0.99, and *ϵ* = 10^−7^ were used in all experiments. The models were trained for 100 epochs with a batch size of 64. We adopted image augmentation to increase the effective size of the training data. Specifically, we first zero-padded the image to a size of 280 × 280 and then cropped the image back to 256 × 256 with the center at a random location in the padded image. For the models that included stacked self-attention layers, we used self-attention layers with the number of heads of one, hidden dimension size of 128, and hidden dimension size of the feedforward layer of 512.

#### Evaluation

We performed 5 random training-validation-test data splittings and reported the mean and standard deviation of the classification accuracies and F1-scores obtained from each method. Since all the methods were trained to predict the probability of having MCI, *p*, for each input, we needed to convert the model prediction into a diagnostic label (i.e., hard label) so that we could compute the accuracy and F1-score meaningfully. In this case, we categorized all the subjects with *p* ≥ 0.5 as MCI patients and *p* < 0.5 as healthy controls. We also reported the AUCs for all the models under consideration.

We also assessed the ability of the proposed method to provide a visual explanation to support its diagnostic decision by comparing the heat maps generated by the proposed model to those generated by the multi-input VGG16 model with Grad-CAM [19], which is one of the most commonly used methods for visual explanation, based on two metrics: (1) the interpretability scores given by 3 experts and (2) the IoU between the heat maps obtained from each method and the corresponding ground truth ROIs.

For each subject, we obtained the heat maps from both the proposed method and the multi-input VGG16 model. Then, we displayed them side-by-side and separately asked 1 neurologist and 2 licensed neuropsychologists to give scores between 1 and 5 to each set of heat maps (1 being the worst and 5 being the best in terms of providing a visual explanation that aligned with their experience and knowledge). To avoid potential bias, we randomly shuffled the display locations (left vs. right) in a way that the heat maps of the proposed method were displayed on the left or the right of the VGG16 model with equal probability. We ensured that all the evaluators had sufficient clinical experience in testing and evaluating these drawing tasks while still allowing them to rate the interpretability of the heat map results using their own judgments. The rationale is that the interpretability of the heat map can be evaluated in more than one perspective, and our proposed model should generally perform better than the Grad-CAM model across multiple perspectives.

In addition to the rating provided by the experts, the interpretability of the heat maps was assessed based on the IoUs between the heat maps and ground truth ROIs, where the IoU between two arbitrary shapes *A* and *B* is computed as $IoU\left(A,B\right)=\frac{\left|A\cap B\right|}{\left|A\cup B\right|}$. Prior to computing IoUs, each heat map was converted into a binary matrix by assigning 1 to the top *k* percent of all pixels with the highest values and setting the remaining pixels to 0. As shown in Fig. 1, two types of ROIs were used as the ground truth ROIs in our evaluations: whole-drawing ROIs and expert ROIs.

For the whole-drawing ROIs, the goal was to check if the heat maps highlighted the regions in the vicinity of the regions drawn by the human subjects. For each image, we defined an initial ROI as the smallest region with a simple shape (e.g., an ellipse, a circle, and a polygon) that enclosed all the non-zero pixels in the image. Then, we enlarged the resulting ROI by a few pixels and used it as the ground truth ROI. Under this ROI type, the heat maps that do not highlight regions far away from the regions drawn will achieve relatively high IoU. For example, for the clock drawing test, if a heat map focuses on the locations outside the clock, the heat map is considered to have low visual explainability under this metric since it provides no visual cues to substantiate the model’s prediction. Note that the heat maps that yield higher IoU are not necessarily more interpretable by clinicians since the highlighted pixels could be randomly moved without decreasing the IoU as long as they are still located inside the ROIs.

For the expert ROIs, the ground truth ROIs were constructed based on the assumption that a good heat map should capture the presence of the paths that should not be drawn and/or the absence of the paths that should be drawn. So, the ground truth ROI for each image contains the regions that include such unusual paths as confirmed by an experienced clinician. This metric more closely resembles how clinicians would interpret the results in everyday practices. While a heat map that highlights unusual paths in the image would achieve high IoU with respect to the expert ROIs, a model that generates a heat map that focuses only on the usual paths (i.e., paths typically drawn by healthy aging controls) would achieve lower IoU. Therefore, IoUs with the expert ROIs should also be used and interpreted with caution.

## Data Availability

The data used in this research are publicly available on GitHub at https://github.com/cccnlab/MCI-multiple-drawings.
